# Cognitive Impairments in *LRRK2*-Related Parkinson's Disease: A Study in Chinese Individuals

**DOI:** 10.1155/2015/621873

**Published:** 2015-08-06

**Authors:** Yifan Zheng, Zhong Pei, Yanmei Liu, Hongyan Zhou, Wenbiao Xian, Yingying Fang, Ling Chen, Qi Wu

**Affiliations:** Department of Neurology, The First Affiliated Hospital, Sun Yat-sen University, Guangzhou 510080, China

## Abstract

*Background*. *LRRK2* S1647T has been identified as a polymorphic risk variant for Parkinson's disease (PD) in Chinese individuals. As *LRRK2* is the most common genetic cause for PD, it has drawn great interest regarding whether cognitive impairments in PD are related with *LRRK2*. *Purpose*. This study aimed to explore the effects of *LRRK2* S1647T polymorphism on cognitive function in PD. *Method*. 90 PD patients were randomly recruited. They underwent a series of clinical evaluations and genetic testing for the *LRRK2* S1647T polymorphism. Global intellect and five cognitive domains (language fluency, visuospatial function, attention, memory, and executive function) were compared between S1647T carriers and noncarriers. *Results*. No differences in motor features were found between two groups, but the executive function evaluation showed that Stroop word colour test time (SWCT-TIME) scores were lower in *LRRK2* S1647T carriers than in noncarriers (*P* = 0.017). However, multiple linear regression analysis indicated that the correlation between S1647T polymorphism and SWCT-TIME scores did not reach significant level (*P* = 0.051). *Conclusion*. Our findings suggest that cognitive impairments are not correlated with different *LRRK2* S1647T polymorphisms in Chinese PD individuals.

## 1. Introduction

Although the aetiology of Parkinson's disease (PD) remains unclear, a complex interaction between genetic and environmental factors is generally considered to be its cause [1]. Mutations in the gene encoding for leucine-rich repeat kinase 2 (LRRK2) at the PARK8 locus on chromosome 12q12 may be related with PD.* LRRK2* S1647T has been identified as a risk variant for PD in the Chinese population [2]. Among our PD samples in southern China, 35.7% were heterozygous and 24.4% were homozygous for this allele [3].

Clinically,* LRRK2*-related PD is characterized by motor features consistent with idiopathic PD, including asymmetrical tremor with bradykinesia and rigidity that responds to dopamine replacement [4]. However, nonmotor symptoms, especially those related to cognition, are not well characterized in* LRRK2*-related PD. We previously demonstrated that cognitive impairment is common in the early and middle stages of PD without dementia, and executive function is the most commonly impaired domain in a Chinese population of PD [5]. Mutations in the gene encoding *α*-synuclein (SNCA) have been associated with cognitive impairments in PD, suggesting that genetic factors influence cognition in patients with PD [6]. As* LRRK2* is the most common genetic cause for PD [7], it has drawn great interest concerning whether cognitive impairments in PD are related with* LRRK2* [8]. As the S1647T polymorphism is common in Chinese populations, we performed detailed neuropsychological evaluations of 90 PD patients (45* LRRK2* S1647T carriers and 45 noncarriers) to explore the effect of this polymorphism on cognitive function in PD and to achieve a more extensive clinical characterization of patients with this polymorphism.

## 2. Materials and Methods

The study was carried out in accordance with the Guide for the Experimentation with Humans. The protocol was approved by the Ethics Committee of Sun Yat-sen University, and all subjects provided their written informed consent to participate in the study.

### 2.1. Subjects

PD participants were randomly recruited in the PD clinic and the ward of Department of Neurology at the First Affiliated Hospital, Sun Yat-sen University, China, between November 2008 and April 2010. The diagnosis of PD was made by neurologists using the UK Brain Bank criteria [9]. The study ruled out patients with identified dementia (according to DSM-IV criteria for dementia) and/or definite depression (Hamilton Depression Rating Scale (HAMD) ≥ 20 points) [10], definite anxiety (Hamilton Anxiety Rating Scale (HAMA) ≥ 22 points) [11], color blindness, and deafness. Illiterate patients were also excluded. Finally, a total of 90 PD patients were included in this study. They underwent a series of clinical evaluations and genetic testing for the* LRRK2* S1647T polymorphism after providing informed consent.

### 2.2. Gene Polymorphism Analysis

DNA was extracted from a peripheral venous blood sample using standard procedures, and* LRRK2* S1647T polymorphisms were identified with polymerase chain reaction-restriction fragment length polymorphism analysis.

### 2.3. Clinical Assessment

All cases were in “on” condition when the evaluation was conducted. They were under stable dopaminergic therapy and did not require booster doses of L-DOPA or dopamine agonists. Clinical and demographic data were collected using standardized case report forms that included the Unified Parkinson's Disease Rating Scale (UPDRS) [12] and Hoehn and Yahr Scale [13]. The neuropsychological battery was performed in a single session, including the Mini-Mental State Examination (MMSE, used to assess global mental status) [14]. Five cognitive domains involving language fluency, visuospatial function, memory, attention, and executive function were evaluated by the animal fluency test (AFT) [15], clock-drawing test (CDT) [16], Rey auditory verbal learning test (AVLT) [17], Wechsler adult intelligence scale digit span task (DST) [18], and Stroop word colour test (SWCT) [19]. Both SWCT-TIME and SWCT-ERROR scores were calculated by scores in the incongruous Stroop condition minus scores in the basal condition, so that the presence of basal differences in performing the SWCT test between the two groups can be excluded. A single rater who was blind to the clinical characteristics and genotypes of the subjects performed the cognitive evaluations.

### 2.4. Statistical Analysis

SPSS 17.0 software (SPSS Inc., Chicago, IL, USA) was used. Comparisons between* LRRK2* S1647T carriers and noncarriers were computed using chi-square and *t*-tests. A multiple linear regression model was used to explore the risk factors of cognitive impairments. Independent factors that had a possible association with cognitive impairments were entered into a multiple linear regression model, with the scores of cognitive assessments as the dependent variable. A value of *P* < 0.05 was considered significant.

## 3. Results

### 3.1. General Demographic and Clinical Assessment

The gene polymorphism analysis showed that our cohort included 45 carriers and 45 noncarriers of* LRRK2* S1647T. As shown in Table 1, there were no marked differences in general demographic data including sex, age, and education between carriers and noncarriers of* LRRK2* S1647T. We also did not identify differences between the two groups regarding clinical data such as age of onset, duration of illness, PD symptoms and severity, or L-DOPA dose.

### 3.2. Neuropsychological Assessment

Neuropsychological examinations did not reveal remarkable differences in language fluency (AFT), visuospatial function (CDT), attention (DST), memory (AVLT), or global cognitive function (MMSE) between* LRRK2* S1647T carriers and noncarriers, but there was a significant difference in executive function (SWT-TIME scores) between the two groups (Table 2). A significant difference (*P* = 0.049) was also found for HAMD, with lower scores in the S1647T carriers, although the patients with definite depression have been excluded (Table 2).

### 3.3. Analysis of Multiple Factors for SWCT-TIME Scores

Multiple linear regression analysis indicated that the correlation between S1647T polymorphism and SWCT-TIME scores did not reach significant level (*P* = 0.051). In addition, the possible risk factors for cognitive impairments were all found not correlated with the SWCT-TIME scores significantly after multiple linear regression analysis (Table 3).

## 4. Discussion

*LRRK2* mutations were first identified as a cause of autosomal-dominant PD by Zimprich et al. in 2004 [20]; this finding had an instant, significant, and lasting impact on the understanding of the genetic basis of PD. Since then, additional* LRRK2* polymorphisms in different loci have been reported [2, 21]. Although they are distributed worldwide, the effects of* LRRK2* polymorphisms may vary in different populations. The* LRRK2* S1647T polymorphism is found in Chinese population, but its influences on the clinical features of PD remain to be elucidated.

To answer the question of whether the* LRRK2* S1647T polymorphism affects PD symptoms in Chinese subjects, we compared the core features of PD between two groups of patients: 45* LRRK2* S1647T carriers and 45 noncarriers. We did not identify any differences in motor symptoms between the two groups, which is consistent with a previous report that motor features in patients with* LRRK2*-related PD were similar to those with idiopathic PD [4].

In this study, MMSE was used as the global cognitive test but not MOCA which overlaps the animal fluency test and clock-drawing test in the following assessments. The result did not reveal significant differences in MMSE scores between* LRRK2* S1647T carriers and noncarriers. Surprisingly, the SWCT-TIME scores of* LRRK2* S1647T carriers were significantly lower than those of* LRRK2* S1647T noncarriers, but there were no differences in the scores of other scales evaluating other cognitive domains such as language fluency, visuospatial function, memory, or attention. The risk factors for cognitive impairment, such as age, education, disease severity, and anticholinergic use, were also not significantly different between the two groups. Seemingly this finding suggests that executive function impairment is less severe in* LRRK2* S1647T carriers. To be cautious, we conducted a multiple linear regression analysis and found that S1647T polymorphism was not the predictor of SWCT-TIME scores. The significant differences found by *t*-tests between* LRRK2* S1647T carriers and noncarriers may be due to the fact that carriers were younger than noncarriers, even though no significant differences emerge between two groups. Another factor which could help to explain the better SWCT-TIME scores of carriers was the significantly lower HAMD scores of carriers, for the exclusion criteria of HAMD ≥ 20 points only excluded the patients with definite depression but those with possible depression were not excluded yet. It is well documented that cognitive scores can be influenced by depression state [22]. However, we cannot gain a conclusion that carriers are less depressive than noncarriers because the exclusion of patients with definite depression made the result not comprehensive.

Although there is no existing study regarding the correlation of nonmotor features and* LRRK2* S1647T, some similar reports on relationships between cognitive impairment and* LRRK2* polymorphisms in other loci reached different conclusions. In 2004, Paisán-Ruíz et al. [23] studied a small series of* LRRK2* R1396G patients and reported that there was lower prevalence of cognitive impairments in* LRRK2-*related PD compared to subjects with idiopathic PD. In a multicentre study with large samples from across the world, Healy et al. [24] reported that cognition changes in patients with* LRRK2*-related PD were milder than those in subjects with idiopathic PD. However, the large samples did not include Chinese population. Estanga et al. [25] performed a detailed cognitive study of 60 Spanish PD patients and found that the neuropsychological performance of carriers with another* LRRK2* variant (R1441G) was similar to that of* LRRK2* R1441G noncarriers. The different conclusions among these studies might be due to different loci and different samples. Our study is the unique study to investigate the relationship between the particular polymorphism S1647T and cognitive function in Chinese PD population and it may contribute to the improvement of evaluation in worldwide PD population.

## 5. Conclusion

In summary, cognitive function seems to be similar among PD patients with different* LRRK2* S1647T polymorphisms. However, these findings need to be confirmed by prospective studies that assess larger samples. Another limitation of this study was that it did not include a demographically matched control group. As a result, we did not have access to normative control data for the assessments and therefore cannot compare the level of cognitive difficulty experienced by the research subjects with control subjects. Further comparative studies between PD patients and healthy individuals with or without this polymorphism are needed to better examine its effect on cognitive function.

## Figures and Tables

**Table 1:** General demographic and clinical characteristics of patients with PD.

	S1647T carriers	S1647T noncarriers	*P* (*χ* ^2^/*t*) values
Sex ratio (M/F)	30/15	25/20	0.280 (1.169)
Age (years)	60.79 ± 10.19	64.04 ± 8.17	0.098 (−1.672)
Education (years)	11.49 ± 4.22	11.31 ± 3.89	0.836 (0.208)
Age at PD onset (years)	55.51 ± 10.14	59.01 ± 8.04	0.073 (−1.814)
Disease duration (years)	5.29 ± 3.96	4.96 ± 3.86	0.691 (0.399)
Onset side (right/left)	34/11	36/9	0.612 (0.257)
Symptom at PD onset (tremor/not tremor)	27/18	26/19	0.830 (0.046)
Axial symptom (yes/no)	29/16	23/22	0.200 (1.640)
UPDRS III	23.34 ± 9.88	23.56 ± 12.71	0.930 (−0.088)
Hoehn and Yahr	2.02 ± 0.72	2.02 ± 0.83	1.000 (0)
L-DOPA dose (mg/d)	379.68 ± 264.27	400.39 ± 222.44	0.689 (−0.402)
Anticholinergic use (yes/no)	32/13	35/10	0.468 (0.526)

**Table 2:** Cognitive assessment of S1647T carriers and noncarriers with PD.

Variables	S1647T carriers (*n* = 45)	S1647T noncarriers (*n* = 45)	*P* (*t*) values
MMSE	27.78 ± 1.58	28.20 ± 1.83	0.244 (−1.172)
AVLT (immediate)	40.29 ± 10.31	40.07 ± 11.12	0.922 (0.098)
AVLT (20 min)	8.87 ± 2.84	8.73 ± 3.48	0.843 (0.199)
SWCT-TIME	44.20 ± 23.06	56.98 ± 26.64	0.017 (−2.433)
SWCT-ERROR	4.67 ± 3.00	5.60 ± 4.63	0.172 (−1.377)
Animal fluency test	16.71 ± 4.25	15.91 ± 5.20	0.426 (0.800)
Clock-drawing test	3.82 ± 0.44	3.89 ± 0.38	0.446 (−0.765)
Digit span task	12.73 ± 1.95	12.73 ± 1.88	1.000 (0)
HAMD	3.44 ± 3.23	5.11 ± 4.58	0.049 (−1.994)
HAMA	3.44 ± 2.58	4.11 ± 3.41	0.298 (−1.046)

**Table 3:** Predictors of SWCT-TIME scores (multiple linear regression analysis).

Variable	*P* value	Beta value	95% CI	
			Lower	Upper
S1647T polymorphism	0.051	0.213	−0.044	21.740
Age (years)	0.244	−3.460	−25.608	6.621
Education (years)	0.776	−0.030	−1.496	1.121
Age at PD onset (years)	0.220	3.615	−6.105	26.072
Disease duration (years)	0.214	1.528	−5.904	26.003
UPDRS III	0.087	0.217	−0.072	1.053
Hoehn and Yahr	0.883	−0.020	−9.811	8.454
L-DOPA dose (mg/d)	0.344	0.129	−0.015	0.042
HAMA	0.901	0.017	−2.144	2.431
HAMD	0.644	0.065	−1.358	2.183
